# Polyvinylpyrrolidone—Alginate—Carbonate Hydroxyapatite Porous Composites for Dental Applications

**DOI:** 10.3390/ma16124478

**Published:** 2023-06-20

**Authors:** Anna A. Forysenkova, Inna V. Fadeeva, Dina V. Deyneko, Alevtina N. Gosteva, Georgy V. Mamin, Darya V. Shurtakova, Galina A. Davydova, Viktoriya G. Yankova, Iulian V. Antoniac, Julietta V. Rau

**Affiliations:** 1 A.A. Baikov Institute of Metallurgy and Material Science RAS, Leninsky, 49, 119334 Moscow, Russia; aforysenkova@gmail.com (A.A.F.); fadeeva_inna@mail.ru (I.V.F.); 2 Chemistry Department, Lomonosov Moscow State University, Leninskie Gory 1, 119991 Moscow, Russia; deynekomsu@gmail.com; 3 Laboratory of Arctic Mineralogy and Material Sciences, Kola Science Centre RAS, 14 Fersman Str., 184209 Apatity, Russia; 4 Tananaev Institute of Chemistry, Kola Science Centre RAS, Akademgorodok 26A, 184209 Apatity, Russia; angosteva@list.ru; 5 Institute of Physics, Kazan Federal University, Kremlevskaya 18, 420008 Kazan, Russia; georgemamin@gmail.com (G.V.M.); darja-shurtakva@mail.ru (D.V.S.); 6 Institute of Theoretical and Experimental Biophysics of RAS, Institutskaya 3, Puschino, 142290 Moscow, Russia; davidova_g@mail.ru; 7 Department of Analytical, Physical and Colloid Chemistry, Institute of Pharmacy, I.M. Sechenov First Moscow State Medical University, Trubetskaya 8, Build. 2, 119991 Moscow, Russia; yankova_v_g@staff.sechenov.ru; 8 Faculty of Materials Science and Engineering, University Politehnica of Bucharest, 313 Splaiul Independentei Street, District 6, 060042 Bucharest, Romania; antoniac.iulian@gmail.com; 9 Academy of Romanian Scientists, 54 Splaiul Independentei Street, District 5, 050094 Bucharest, Romania; 10 Istituto di Struttura della Materia, Consiglio Nazionale delle Ricerche (ISM-CNR), Via del Fosso del Cavaliere, 100, 00133 Rome, Italy

**Keywords:** composite, hydroxyapatite, carbonate hydroxyapatite, alginate, polyvinylpyrrolidone

## Abstract

An alternative approach for the currently used replacement therapy in dentistry is to apply materials that restore tooth tissue. Among them, composites, based on biopolymers with calcium phosphates, and cells can be applied. In the present work, a composite based on polyvinylpyrrolidone (PVP) and alginate (Alg) with carbonate hydroxyapatite (CHA) was prepared and characterized. The composite was investigated by X-ray diffraction, infrared spectroscopy, electron paramagnetic resonance (EPR) and scanning electron microscopy methods, and the microstructure, porosity, and swelling properties of the material were described. In vitro studies included the MTT test using mouse fibroblasts, and adhesion and survivability tests with human dental pulp stem cells (DPSC). The mineral component of the composite corresponded to CHA with an admixture of amorphous calcium phosphate. The presence of a bond between the polymer matrix and CHA particles was shown by EPR. The structure of the material was represented by micro- (30–190 μm) and nano-pores (average 8.71 ± 4.15 nm). The swelling measurements attested that CHA addition increased the polymer matrix hydrophilicity by 200%. *In vitro* studies demonstrated the biocompatibility of PVP-Alg-CHA (95 ± 5% cell viability), and DPSC located inside the pores. It was concluded that the PVP-Alg-CHA porous composite is promising for dentistry applications.

## 1. Introduction

One of most common oral diseases is dental caries, which affects more than 90% of the population in Western countries [1,2]. Starting as enamel damage, caries eventually affects the soft tissues of the tooth (pulp), which leads to inflammation and necrosis [3]. In the most cases, the treatment of caries consists of the removal of necrotic tissues and their replacement with various restorative materials [3]. For these purposes, cements and ceramic materials based on calcium phosphates (CaP) are widely used [4]; in particular, hydroxyapatite (HA, Ca_10_(PO_4_)_6_(OH)_2_)) is used due to its similarity with the mineral component of the hard tissues of teeth [5,6].

Recently, the development of new methods of treatment that take into account the tissue structure of teeth, namely the combination of mineralized and non-mineralized components, dentin and pulp, has become relevant [1]. This approach implies the restoration of tooth function and the extension to the period during which teeth appear healthy [7]. In perspective, these issues can be solved by porous composites based on biopolymers with CaP [6,8].

To prepare composites, a variety of polymers, both synthetic and natural, can be used, including polylactic acid derivatives, polyacrylate, polyvinyl alcohol, collagen, chitosan, alginate (Alg), etc. [9]. These polymers have their own advantages and disadvantages. In particular, synthetic polymers are bioinert and non-immunogenic, but do not undergo bioresorption [10]. Natural polymers are capable of bioresorption, but, as a rule, have lower strength characteristics [11]. The optimal solution is to combine various polymers to obtain materials with unique properties.

CaP is available in the form of cements [12], granules [13] or powders [14]. CaP may contain substituting ions [15,16,17] to impart osteoinductive (Sr) [18] or antibacterial (Cu, Zn, Ag, Mn, Gd) [19,20,21,22,23] properties. For dental applications, carbonate hydroxyapatite (CHA, Ca_10_(PO_4_)_6_(CO_3_)_0.5_(OH) is a good choice, because of its similar composition to the mineral component of dentin [24].

Porous structures are required for better bio-integration and angiogenesis [25,26]. They can be obtained by freeze-drying of gels [27]. In this way, such composite materials as collagen-HA [28], chitosan-HA [29], and gelatine-HA have been generated [30]. Therefore, gel-forming natural or synthetic hydrophilic polymers need to be employed [31], such as alginate and polyvinylpyrrolidone (polyvidone, povidone) (PVP), described in [11]. Alginate is a natural polysaccharide that forms viscous gels [32]. PVP is a synthetic polymer that is highly soluble in water [33]. Separately, these polymers as well as composites based on them have been studied [34,35]. However, literature data on their mixtures possessing unique properties, as well as composite materials based on them, are scarce. Previously, we reported film materials based on PVP-Alg-HA [14], with HA prepared *in situ.*

In the present work, a composite based on the mixture of PVP and Alg with CHA obtained by gel freeze-drying was developed. CHA was synthesized *ex situ*. The composition, structure and physico-chemical properties of the material were studied by X-ray diffraction (XRD), Fourier transform infrared spectroscopy (FT-IR), electron paramagnetic resonance (EPR), and scanning electron microscopy (SEM) methods. The porosity and swelling properties of the material were investigated. Its biocompatibility was assessed by the MTT test, applying the NCTC clone L-929 fibroblast of mouse subcutaneous connective tissue. The adhesion and viability of dental pulp stem cells (DPSC) on the composite surface were investigated, and perspectives for its applications in dentistry were described.

## 2. Materials and Methods

### 2.1. PVP-Alg-CHA Preparation

CHA and HA (prepared for EPR studies described in Section 2.4) were synthesized *ex situ* by precipitation in accordance with Equation (1):

   10Ca(NO_3_)_2_ + 6(NH_4_)_2_HPO_4_ + 0.5(NH_4_)_2_CO_3_ + 7NH_4_OH → → Ca_10_(PO_4_)_6_(CO_3_)_0.5_(OH)↓ + 20NH_4_NO_3_ + 6H_2_O (1)

The resulting suspension was filtered on a Buchner funnel using a vacuum pump and dried at 110 °C to a constant mass.

**Figure 1:**
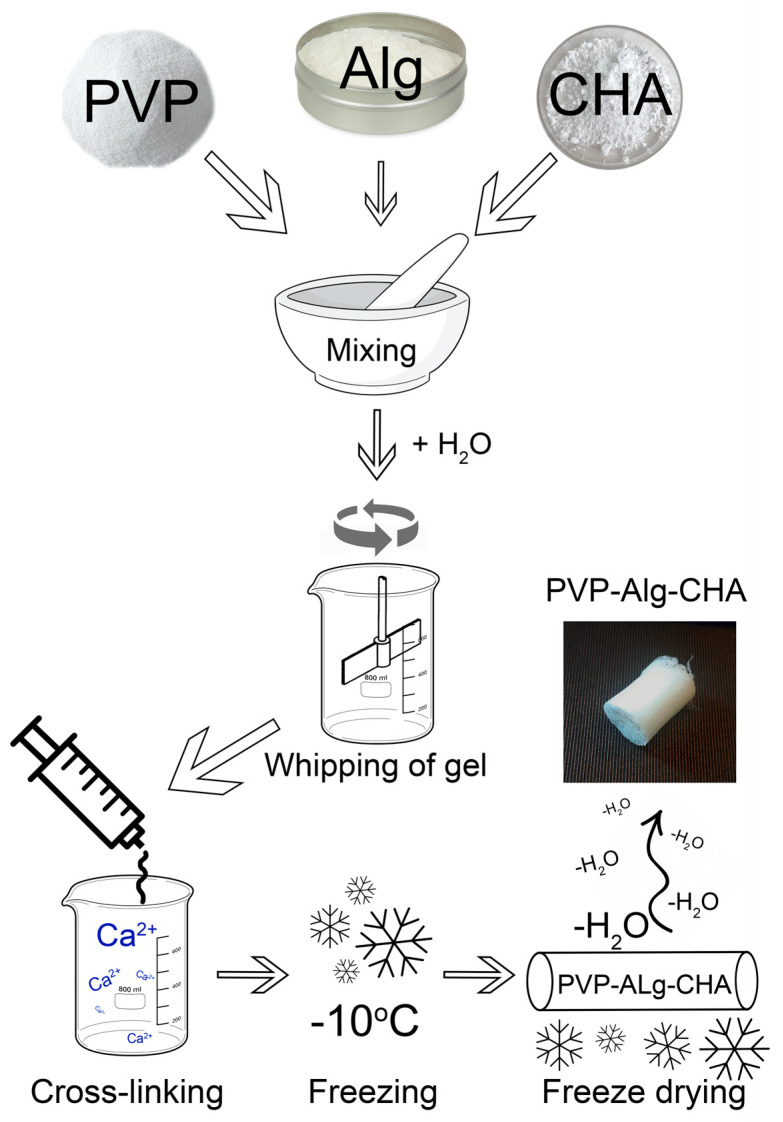
The scheme of PVP-Alg-CHA composite preparation.

To obtain composite materials, CHA powder was milled to ≤50 μm and mixed with powders of PVP (360 kDa, Sigma-Aldrich, St. Louis, MO, USA) and Alg (pure, Reakhim, Moscow, Russia) polymers at a concentration of 5 wt.%. The powder mixture was dissolved in water to obtain a gel with a PVP-Alg-CHA content of 2.5 wt.%. The resulting gel was mixed until a homogeneous mixture was obtained using a top-drive agitator at a speed of 700 rpm. To obtain porous materials, the resulting gel was whipped at a speed of 7000 rpm. The foam obtained was squeezed out through a syringe into a pre-prepared solution of 0.1 mol/L CaCl_2_ (Khimed, Moscow, Russia) for cross-linking of Alg [36]. After 5 min, materials were pulled out from the solution, frozen at a temperature of −10 °C and then dried in an LS-1000 freeze dryer (Prointech, St. Petersburg, Russia). The sample obtained was named PVP-Alg-CHA. The preparation scheme is presented in Figure 1.

### 2.2. XRD Analysis

Powder X-ray diffraction (PXRD) patterns were collected on a Rigaku SmartLab SE diffractometer (Rigaku, Wilmington, MA, USA) with a 3 kW sealed X-ray tube, D/teX Ultra 250 silicon strip detector, vertical type θ-θ geometry, and HyPix-400 (2D HPAD) detector. PXRD data were collected at room temperature in the 2θ range of 3° to 110° with a step interval of 0.02°. PXRD patterns were plotted using the Crystallographica Search-Match (Version 2, 0, 3, 1.) and the PFD#2 database.

### 2.3. FT-IR Spectroscopy

Absorption spectra of the samples were recorded on an FT-803 Fourier spectrometer (Simeks Research and Production Company 2022 Novosibirsk, Russia) in the wavenumber region of 4000–400 cm^−1^, with 1 cm^−1^ spectral resolution. The standard KBr disc method was applied to obtained the spectra.

### 2.4. EPR Spectroscopy

Electron paramagnetic resonance studies were carried out with non-carbonated HA, since the CO_3_^2−^ groups displace the NO_3_^−^ groups (coming from residual impurity) from the HA structure, as discussed in an earlier ref. [37]. The EPR studies were carried out by means of a Bruker Elexsys E580 spectrometer (Bruker, Germany) (X-range, ν = 9.61 GHz) and E680 (W—range, ν = 94 GHz) at 200 and 297 K. In pulse mode, the Khan sequence was used: π/2-τ-π-τ – (electronic spin echo (ESE)); where time π/2 = 64 ns, τ = 248 ns. The EPR spectra were obtained by measuring the integral intensity of the ESE with a continuous extension of the magnetic field B_0_. The samples were subjected to X-ray irradiation for 1 h on the URS-55 device with an absorption dose of 15 kGy at room temperature to create stable radiation centers.

### 2.5. SEM

The microstructure of PVP-Alg-CHA composite was studied by scanning electron microscopy using the TescanVegaII microscope (Tescan, Brno, Czech Republic).

### 2.6. Porosity Measurements

The porosity of the PVP-Alg-CHA composite was determined using a TriStar 3000 porosimeter (Micromeritics, Norcross, GA, USA) by low-temperature nitrogen adsorption [38].

### 2.7. Swelling Measurements

Swelling behavior of the porous PVP-Alg-CHA composite was determined by the change in mass after its immersion in deionized water.

### 2.8. In Vitro Cell Tests

The MTT test was used for evaluation of the cytotoxicity of extracts from the investigated materials. It was carried out using cells of the NCTC clone L-929 fibroblast of mouse subcutaneous connective tissue. The 3 day extracts were prepared in accordance with the requirements of GOST R ISO 10993.12-15 [39].

The adhesion and proliferation of the DPSC was investigated by direct contact, as described earlier in the ref. [14]. The cells were spread on the surface of the test samples and placed in the wells of a 24-well plate with a layer density of 40,000 cm^−2^. The viability of DPSC cells on the surface of the composite was assessed by differentiated fluorescent staining of living and dead cells using of an Axiovert 200 inverted microscope (Zeiss, Jena, Germany). The fluorescent dye SYTO9 turns green after the interaction with DNA and RNA of living and dead cells (λ_ex_ = 450–490 nm, λ_emiss_ = 515–565 nm), and propidium iodide (PI) turns red after the interaction with DNA and RNA of dead cells (λ_ex_ = 546 nm, λ_emiss_ = 575–640 nm).

The statistical analysis of the experimental data on DPSC adhesion was carried out. A set of 24 composite samples was placed in a 24-well plate. The extracts from each well were analyzed, and the mean values and corresponding standard deviations were presented. For the adhesion test, the nuclei of dead cells were stained using the intercalating reagent PI (λ_ex_ = 546 nm, λ_emiss_ = 575–640 nm).

## 3. Results and Discussion

### 3.1. Powder X-ray Diffraction (PXRD) Study

**Figure 2:**
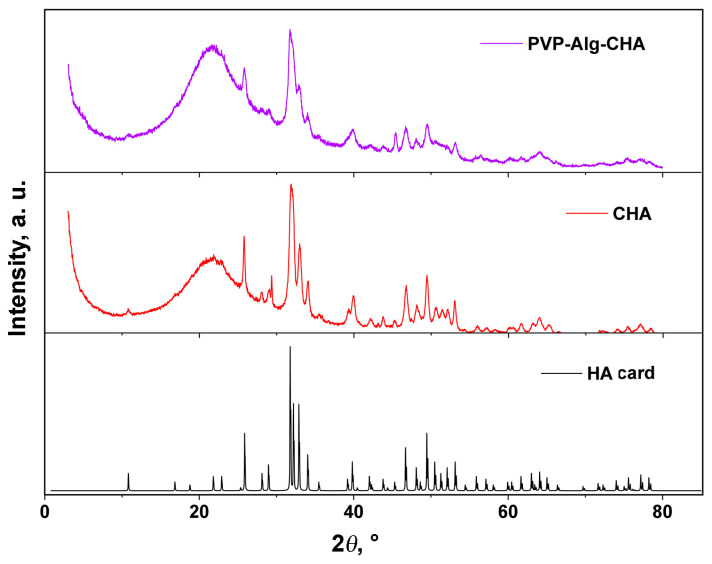
PXRD patterns of CHA obtained by precipitation and of composite PVP-Alg-CHA material, along with HA (card PDF#4 No 00-009-0432).

The PXRD patterns of the CHA sample obtained by precipitation and of the composite material are shown in Figure 2. The profiles of the patterns correspond to poor crystallized HA phase due to the broadening of the reflections. The broad lines on the PXRD patterns at 2θ° = 15–35 were attributed to amorphous calcium phosphate formed according to Equation (2):

*x*Ca^2+^ + *y*HPO_4_^2−^ +*y*OH^−^ + (*n* − *y*)H_2_O → Ca*_x_*(PO_4_)*_y_*·*n*H_2_O↓ (2)

In ref. [40], it was shown that amorphous calcium phosphate transforms into HA upon aging [41].

The broad rage at 2θ° = 3–40 can be attributed to the polymer matrix of the composite (Figure 2, PVP-Alg-CHA pattern). PVP and Alg have some crystallinity and demonstrate broad lines at 10.9 and 21.1 2θ° (PVP) [42,43], and at 13.73 and 21.71 2θ° (Alg) [44].

### 3.2. FT-IR Spectroscopy Investigation

**Figure 3:**
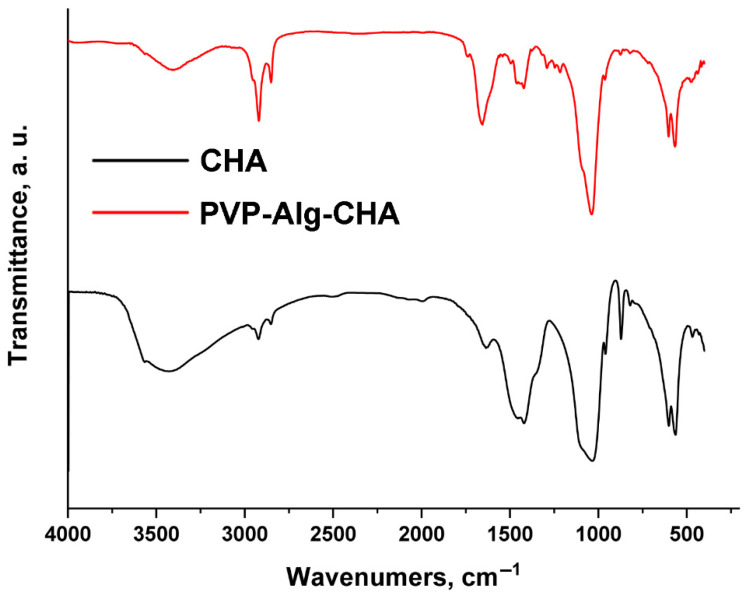
FT-IR spectra of CHA and PVP-Alg-CHA composite.

The chemical composition of the synthesized CHA and PVP-Alg-CHA composites were confirmed by FT-IR spectroscopy (Figure 3). The characteristic oscillation frequencies are given in Table 1.

The OH^−^ ions were present in both the samples. In addition, the banding vibration of water is fixed in CHA and PVP-Alg-CHA. The presence of C−O bond oscillation bands (1420, 1460 cm^−1^) indicated the substitution of PO_4_^3−^–tetrahedra by carbonate ions. This is called the substitution of B-type HA with a low V cell. The absence of the 1550 cm^−1^ band indicated that the OH^−^ groups were not replaced by carbonate ions in the channels of the HA structure. This showed the absence of HA of A-type with a large V cell [45,49]. The presence of amide group in PVP was confirmed by the absorption band at 1738 cm^−1^ [47]. The peak at 1425 cm^−1^ was ascribed to the stretching mode of the C=N partial double bond of PVP [50]. The carbonyl band of PVP appeared at 1663 cm^−1^ [55]. The frequencies of functional groups of Alg (carboxyl group – 1590 cm^−1^, 1411 cm^−1^, ring oxygen 1078 cm^−1^) correspond to calcium alginate [54], which was formed as a result of cross-linking by CaCl_2_.

However, a small amount of NO_3_^−^ ions was detected in CHA. This can be explained by the peculiarity of synthesis by precipitation, often characterized by the presence of a small amount of impurities of initial reagents. A small amount of pyrophosphate was also found in the developed composition of PVP-Alg-CHA [22].

### 3.3. EPR Spectroscopy Investigation

The interaction between the polymer matrix with HA was detected by EPR spectroscopy. The interaction between the phases in the composite is a significant characteristic, described in the ref. [8].

As mentioned in Section 2.4, the use of carbonate HA in the EPR method is not appropriate and, therefore, non-carbonated HA was synthesized *ex situ*, and a composite of PVP-Alg-HA was obtained and investigated by EPR.

**Figure 4:**
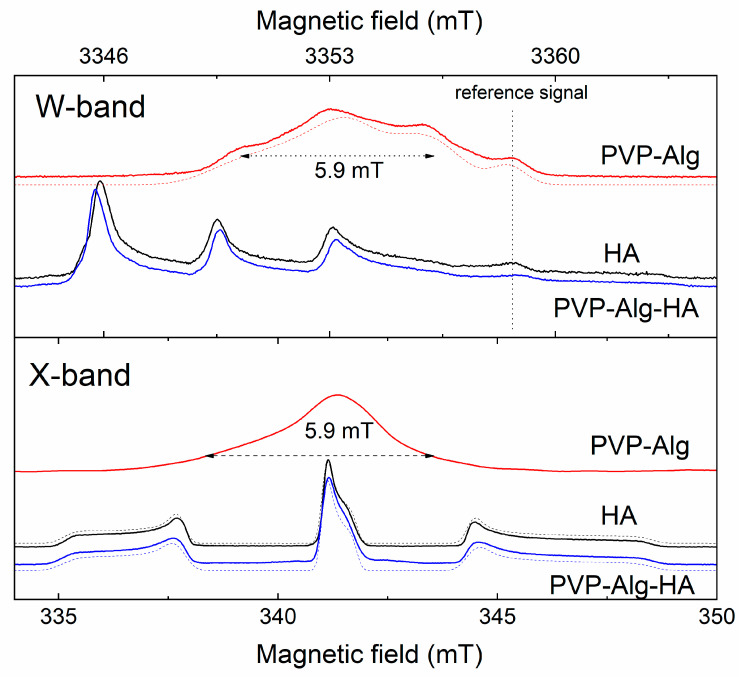
EPR spectra obtained by ESE detection of HA, PVP-Ag, and PVP-Alg-HA samples subjected to X-ray irradiation. W-range at the top; X-range at the bottom. The red lines show the spectra of PVP-Alg, the black lines are referred to HA, and the blue lines to PVP-Alg-HA. The dotted lines show the approximation of the EPR spectra. The approximation parameters are given in Table 2.

The EPR signal was not observed due to the absence of paramagnetic centers (PCs) in the structure of HA and composite samples. After X-ray irradiation, in the HA sample in the X- and W-ranges, three lines of the EPR spectrum characteristic of powders appeared (Figure 4, black lines). The parameters of this spectrum (shown in Table 2) allowed us to confirm the presence of NO_3_^2−^ radicals in the HA structure [56]. PVP-Alg samples after X-ray irradiation were also investigated (Figure 4, red lines). In the W-range, the spectrum of the free radical was observed. In this spectrum, there were three EPR lines, which showed the localization of the unpaired electron on the nitrogen atom of PVP with the constant A_||_ = 106 ± 10 MHz (Table 2). This constant is characteristic of nitrogen radicals in undissolved spin labels. In the X-band, this splitting was hidden in the line-width.

As can be seen from Figure 4 (blue lines), the EPR spectrum of PVP-Alg-HA accounts for the presence of NO_3_^2−^ radicals in HA, and the polymeric mixture components were not observed. This means that the radiation-induced centers in PVP-Alg have a competitive electron trap channel, which is possible only if there is a chemical bond between the components of the composite. In addition, mixing HA with PVP-Alg somewhat changed the hyperfine interaction constants A_⊥_ and A_||_ upwards, and also increased the distribution of the constants ΔA_⊥_ and ΔA_||_ (Table 2). Analysis of changes in A_⊥_ and A_||_ showed that the isotropic part of the hyperfine interaction increased by 3.7 MHz, which corresponds to an increase in the electron density at the nitrogen nucleus in the HA NO_3_^2−^ complex by 2%. It can be assumed that, when HA is added *ex situ*, PVP-Alg molecules form a positively charged layer around the HA particles with the formation of a chemical bond, which increases the electron density in its near-surface layer. Due to electrical neutrality, the charge on the outer surface of the PVP-Alg shell will be negative.

It should be noted that in our previous work [14], the interaction between PVP and HA phases (HA was synthesized *in situ*) was also detected by EPR spectroscopy. In the present work, despite the fact that HA was synthesized *ex situ*, the interaction between the polymer matrix and HA was also confirmed by EPR.

### 3.4. Microstructure

**Figure 5:**
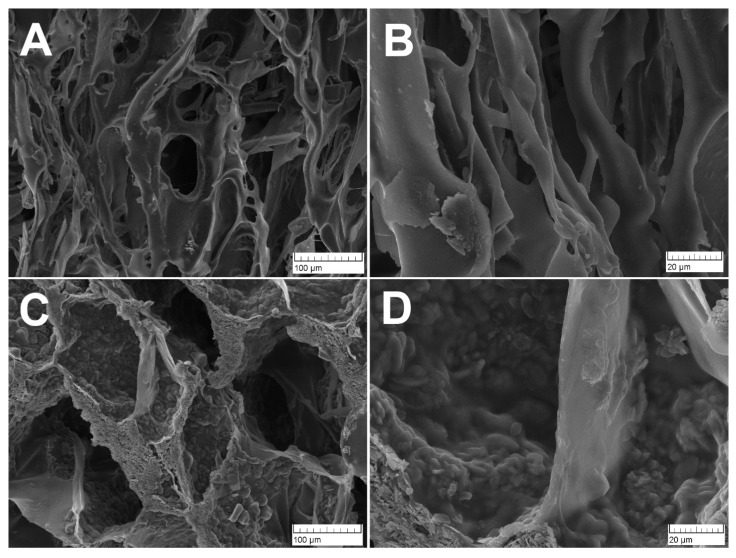
SEM images of PVP-Alg sample (**A**,**B**) and PVP-Alg-CHA sample (**C**,**D**).

SEM images of PVP-Alg and PVP-Alg-CHA samples are presented in Figure 5. The microstructure of PVP-Alg is represented by the large pores with an average size from about 10 to 50 μm, whereas the PVP-Alg-CHA sample has larger pores with an average size from about 30 to 190 μm. This observation is interesting because in the case of film samples of similar composition described in our previous work [14], the introduction of HA led to the reduction of pores. Instead, in this work, the addition of CHA resulted in a significant increase in the pore size. This is likely related to the methods of material preparation. To obtain films, the gels were dried in the air by the evaporation of moisture and spontaneous removal of air bubbles. In contrast, during the preparation of porous composite, the gel whipped into foam was first fixed by partial cross-linking in a calcium solution, and then frozen and dried by freeze-drying. As a result, the porous structure of the gel was saved.

The PVP-polyvinylalcohol(PVA) scaffold characterized by round pores with smooth walls was described in the ref. [57], such morphology being similar to the one obtained in this work for PVP-Alg (see Figure 5A). A similar porous composite structure, such as the one presented in Figure 5C, was observed for the Alg-HA scaffold frozen at −10 °C, as described in ref. [58]. There is a different effect of HA on the size and distribution of the pores. In ref. [57], the increase in HA content synthesized *in situ* (1.5–4.5 wt.%) led to a decrease in the pore size, which was explained by agglomeration of HA particles and their heterogeneous distribution in the gel. In ref. [59], a scaffold made of pure alginate obtained by freeze-drying was characterized by large pores in the order of 500 μm. The addition of HA (6–10 wt.%) also led to a reduction in the pore size to 200–300 μm, whereas in ref. [58], the addition of HA (25 and 50 wt.%) to an alginate-based scaffold did not lead to changes in pore characteristics. It is worth emphasizing here that in the present work, the CHA addition to PVP-Alg led to an increase in pore size. Thus, it is likely that the pore size parameter is influenced by many different factors, including synthesis conditions, etc.

### 3.5. Porosity

**Figure 6:**
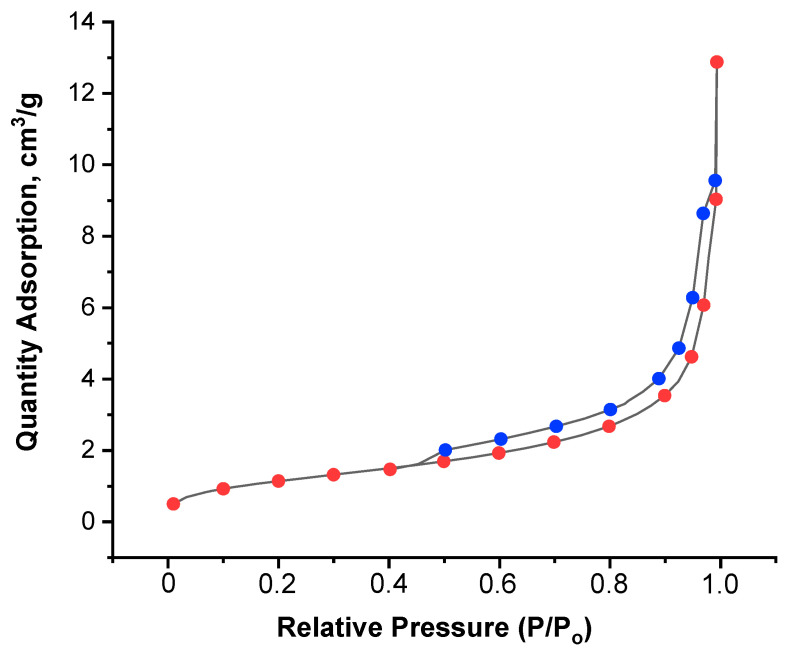
Isotherms of N_2_ adsorption (red dotes) and desorption (blue dotes) on PVP-Alg-CHA.

The porosity data obtained by the BET method are presented in Figure 6 and Table 3. The presence of a hysteresis loop with a characteristic S-shape (Figure 6) indicates the presence of slit-like micropores in the material, which is confirmed by the SEM data (Figure 6). The BET method allowed us to determine the nanoporosity of the developed composite material in terms of total pore volume, average diameter, and specific surface area (Table 3). It should be mentioned that the presence of nanopores can be useful for drug loading [60].

The formation of nano-pores was influenced by the composition of the gel, namely by the polymers forming it. The nanoporosity of an alginate scaffold is affected by the method of alginate cross-linking, as shown in ref. [61]. The nanopores of freeze-dried alginate gel were preserved when the gel was cross-linked with calcium in the solution before freezing, in contrast to the cross-linking of the already dried scaffold (in this latter case, the pores were not preserved) [61]. Thus, the method we have chosen for obtaining the composite—gel cross-linking before freezing allowed us to preserve the nanoporous structure.

### 3.6. Swelling Behavior

**Figure 7:**
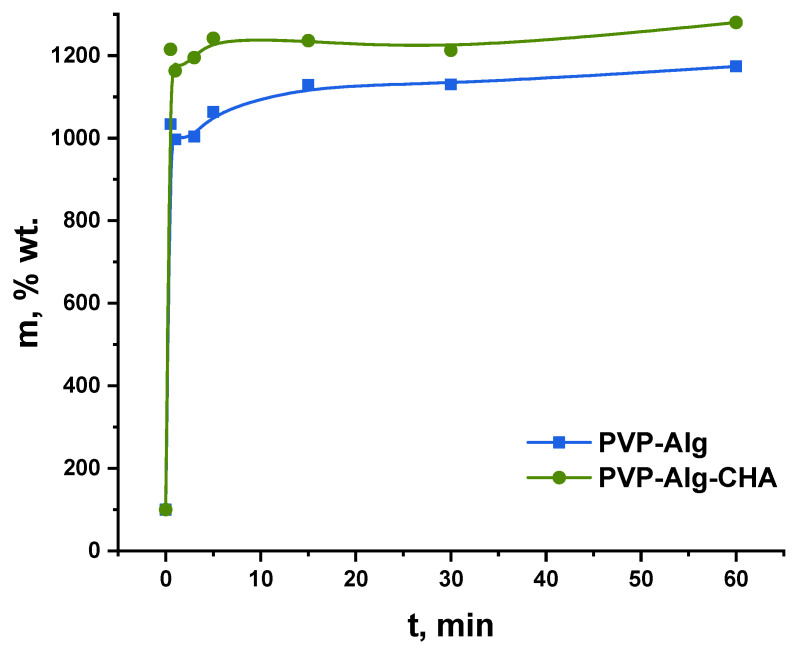
Swelling curves of PVP-Alg and PVP-Alg-CHA in water.

In our previous study [14], it was shown that the introduction of HA led to the reduction of porosity and, consequently, this also reduced the swelling properties of PVP-Alg-HA films. In the case of the porous composite samples developed in this work, a different result was obtained. The swelling curves of PVP-Alg and PVP-Alg-CHA are shown in Figure 7. As can be seen, the degree of swelling was lower for the sample without CHA. This experimental result can be explained by the fact that CHA contains hydrophilic OH^-^ groups in its structure, which are able to bind water through hydrogen bonds. Thus, the introduction of CHA leads to an increase in the degree of swelling of the composite with porous structure. In ref. [57], the swelling of PVP-PVA-HA scaffolds in water, saline, and dextran solution was investigated. It was shown that the increase in the HA content led to an increase in the swelling rate in water, contrary to saline and dextran solutions [57]. This may confirm our assumption that HA also binds water due to the presence of the OH^-^ groups. Although in ref. [59], a sharp decrease in the swelling degree of the Alg-HA hydrogel was demonstrated upon the addition of HA at a concentration of 2 wt.% or more.

### 3.7. In Vitro Cell Tests

**Figure 8:**
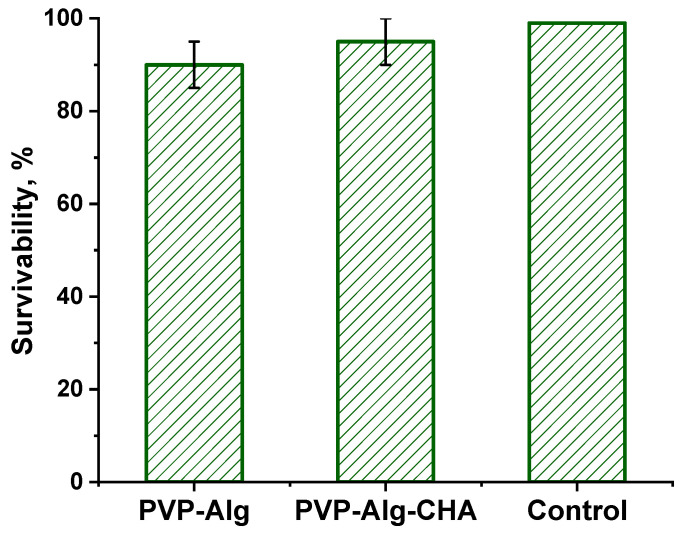
Survivability of NCTC mouse fibroblast cells on PLP-Alg and PVP-Alg-CHA samples.

The MTT test, revealing the biocompatibility of PVP-Alg and PVP-Alg-CHA materials, was carried out using the NCTC clone L-929 of mouse fibroblast cells. As can be seen from the data shown in Figure 8, the cells’ survivability was similar to the control in both samples, and there were no statistically significant differences between PVP-Alg and PVP-Alg-CHA.

**Figure 9:**
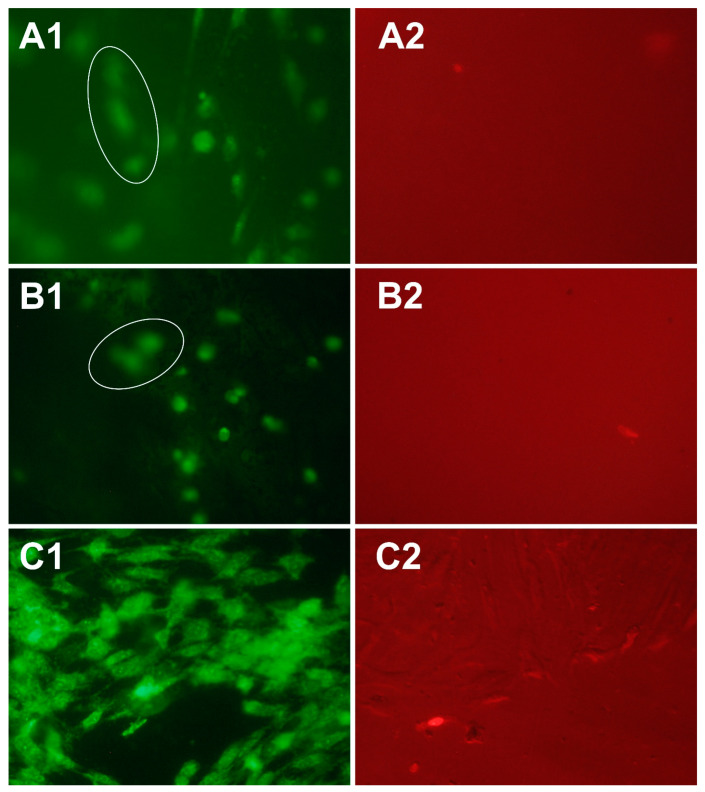
Images of DPSC after 24 h of seeding: green—all cells, red—dead cells. (**A1**,**A2**)—PVP-Alg, (**B1**,**B2**)—PVP-Alg-CHA, (**C1**,**C2**)—control.

The images of DPSC on the surface of PVP-Alg and PVP-Alg-CHA samples are presented in Figure 9. It is clearly visible that the cells have a spherical shape, and many of them are out of the focus of the microscope. This is due to the fact that the composite samples are porous, and the cells were located in the pores (white circles on Figure 9(A1,B1)). The spherical shape of the cells indicated a low adhesion of the cells to the surface of the materials [62]. This property of alginate and PVP, like of many other polymers, is useful for creation of anti-adhesive devices that prevent scarring of wounds and promote normal healing [63]. In the context of this work, this property can also be useful. Low adhesion of the material can prevent fibrosis and improper tissue fusion, and CHA can serve as a material for the functioning of osteoclasts and the growth of new bone tissue. Cell location in the pores of the samples can be also useful from the point of view of the bio-integration of the material.

**Figure 10:**
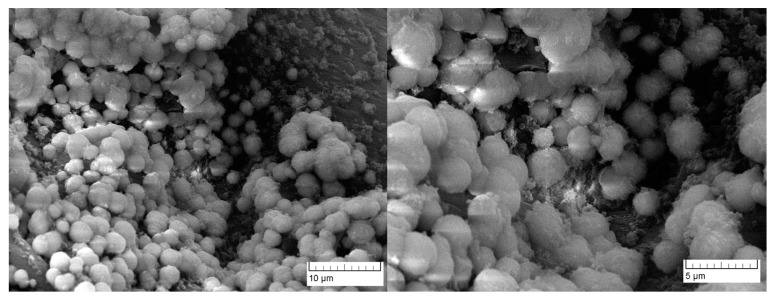
SEM images of DPSC spheroids on the PVP-Alg-CHA surface.

Cell spheroids shown in Figure 10 were also observed on the surface of porous Alg-HA composite reported in ref. [58]. It can be seen that the cells are located inside the pores (Figure 10). The growth of the cell population was associated with an increase in HA content [58]. This effect was also confirmed in our previous research on the biocompatibility of PVP-Alg and PVP-Alg-HA film materials [14].

The developed PVP-Alg-CHA composite has all the necessary characteristics for a possible application in dentistry to eliminate the effects of caries. Since PVP-Alg-CHA is a bulk scaffold, it is supposed to be used in the treatment of deep caries of the posterior teeth [64], but can also be used for anteriors [65]. Since the composite is white, aesthetic problems should not arise.

CHA present in the composite is not only very close to the mineral composition of dentin, but also has a higher resorbability with respect to HA [66]. Alginate, cross-linked with calcium ions, could be an additional source of calcium ions for bone tissue cells and, therefore, also promotes mineralization [67]. In addition, the cross-linked alginate allows the scaffold microstructure to be preserved when in contact with liquids. This, in turn, is necessary for the biointegration of the material namely, for the colonization of cells [68]. A porous microstructure is essential for teeth vascularization [69]. The PVP in the scaffold reduces its mechanical stiffness and, being non-crosslinked, will be removed faster than the alginate mesh, creating more space for tissue growth. The hydrophilic nature of polymers prevents cell adhesion [68,70]; their attachment will proceed gradually as the matrix dissolves and mineralizes. Due to the high swelling capacity of the composite, the scaffold can be used as a local hemostatic agent [71]. In this case, the material will be loaded with blood proteins necessary for cell proliferation and tissue repair. Finally, the nanoporosity of the scaffold can be used for drug delivery.

## 4. Conclusions

Porous composite material based on the mixture of PVP and Alg polymers with CHA, prepared by freeze-drying of the gel, was obtained and characterized. The mineral component of the developed composite was mainly represented by the B-type carbonate hydroxyapatite, according to XRD and IR spectroscopy data. The microstructure of PVP-Alg-CHA was characterized by large pores of about 30–190 μm, and the swelling was 1200 wt.%. The BET method showed that along with micropores, the composite has nanopores, the total volume of which is 0.0094 cm^3^/g, and average size is 8.71 ± 4.15 nm. MTT test studies showed that the developed composite material is biocompatible. The DPSC seeded on the surface of the composite penetrated into its pores, while maintaining a spherical shape.

It can be concluded that the combination of material properties (phase composition, porous microstructure, swelling, nanoporosity, and cell integration) allows us to consider it as a promising material for caries treatment and regenerative therapy in dentistry.

## Figures and Tables

**Table 1:** Vibration modes of CHA and PVP-Alg-CHA composite in the FT-IR spectra.

Assignment	Frequencies, cm^−1^	
	CHA	PVP-Alg-CHA
ν_as_[OH^−^] + ν_s_[OH^−^]	3562	3544
bending vibration [OH^−^]	3399	3378
ν_2_[CO_3_^2−^]	2922	*2916*
ν_as_[CH_2_]		*2916*, 2942
ν_s_[CH_2_]		2847
amide I (C=O ν-s with molecular bond δ)		1738
δ[CH_2_]		*1425*, *1466*
ω[CH_2_]		1323, 1367
τ[CH_2_]		1250, 1289
bending vibration ν_4_(O–P–O) in [PO_4_^3−^]	565, *603*	563, *606*
ν_3as_[P–O]	1034	*1038*
ν[C−C]		*1038*
ν_2_[PO_4_^3−^]	433, 470	417, 438, 469
ν_2_ [NO_3_^−^]	822	826
ν_s_[P−O−P] in P_2_O_7_^4−^		718, 1211
ν_3_[NO_3_^−^]	1350	1367
bending vibration O–C–O bands in [CO_3_^2−^]	873	*872*
bending vibration [H–O–H] in H_2_O	1636	*1663*
carbonyl band of PVP		*1663*
ν_3as_[C–O] in [CO_3_^2−^] of B-type	1418, 1458	*1425*, *1466*, *1495*
δ[C−N]		*1425*, *1466*, *1495*
ν[C−N]		1289
ν [C–N] partial double bond of PVP		*1425*, *1466*, *1495*
ν_3as_ [PO_4_^3−^]	1103	1092
ν_1s_ and ν_3s_ of [P–O] in PO_4_^3−^	963	959
δ[OH^−^]	*603*	*606*
ρ[C–H_2_]		*872*
ν[C−C] + ρ[CH_2_]		*959*
νa_s_[O−C=O] in COO^−^ of Alg		1590
ν_s_[O−C=O] in COO^−^ of Alg		1411
ν [O−C−O] ring		1078

The intervals of characteristic bands for the [PO_4_^3−^] and [C−C], [CO_3_^2−^] and [CH_2_], [C−N] and [CH_2_], [PO_4_^3−^] and [OH^−^] groups partially overlap with each other [45,46,47,48,49,50,51,52,53,54]. Therefore, it is difficult to make an unambiguous attribution. The authors consider that it is acceptable to have a double identification of characteristic values. Repeated frequencies are italicized.

**Table 2:** EPR spectroscopic parameters of the HA and composite.

Sample	g_⊥_	g_||_	A_⊥_ MHz	A_||_ MHz	ΔA_⊥_ MHz	ΔA_||_ MHz
PVP-Alg	2.0022 (2)	2.0026(2)	38 ± 8	106 ± 10	-	-
HA	2.0011(1)	2.0052(1)	92.4 ± 0.5	186 ± 1	7 ± 1	12 ± 1
PVP-Alg-HA	2.0011(1)	2.0052(1)	93.6 ± 0.5	191 ± 1	13 ± 1	18 ± 1

**Table 3:** BET data of PVP-Alg-CHA.

Total Pore Volume, cm^3^/g	Average Pore Diameter, nm	Specific Surface Area, m²/g
0.0094	8.71 ± 4.15	4.31

## Data Availability

The experimental data on the results reported in this manuscript are available upon a reasonable request to corresponding author.
